# Modeling Dependence Structures for Response Times in a Bayesian Framework

**DOI:** 10.1007/s11336-019-09671-8

**Published:** 2019-05-16

**Authors:** Konrad Klotzke, Jean-Paul Fox

**Affiliations:** University of Twente, P.O. Box 217, 7500 AE Enschede, The Netherlands

**Keywords:** response time modeling, conditional independence, local dependence, testlets, Bayesian marginal modeling, non-informative prior distribution

## Abstract

A multivariate generalization of the log-normal model for response times is proposed within an innovative Bayesian modeling framework. A novel Bayesian Covariance Structure Model (BCSM) is proposed, where the inclusion of random-effect variables is avoided, while their implied dependencies are modeled directly through an additive covariance structure. This makes it possible to jointly model complex dependencies due to for instance the test format (e.g., testlets, complex constructs), time limits, or features of digitally based assessments. A class of conjugate priors is proposed for the random-effect variance parameters in the BCSM framework. They give support to testing the presence of random effects, reduce boundary effects by allowing non-positive (co)variance parameters, and support accurate estimation even for very small true variance parameters. The conjugate priors under the BCSM lead to efficient posterior computation. Bayes factors and the Bayesian Information Criterion are discussed for the purpose of model selection in the new framework. In two simulation studies, a satisfying performance of the MCMC algorithm and of the Bayes factor is shown. In comparison with parameter expansion through a half-Cauchy prior, estimates of variance parameters close to zero show no bias and undercoverage of credible intervals is avoided. An empirical example showcases the utility of the BCSM for response times to test the influence of item presentation formats on the test performance of students in a Latin square experimental design.

## Introduction

In various research settings, it is of interest to make inferences about the effect of a treatment or experimental condition on a certain population. For example, two randomly sampled groups from the same population of students may be presented the same literacy test in different forms: The first group takes a traditional pencil-and-paper test, and the second group takes the computer-based counterpart. In that context, a researcher may want to gain insight into the differential functioning of items, or the test as a whole, across the two test forms. In other words, the focus lies on making inferences about the difference in performance between the two testing conditions, not on assessing the individuals’ proficiency in reading and writing. A marginal model is appropriate when inferences about population-averages (e.g., comparing means or (co)variances across groups) are the goal of research (Diggle, Heagerty, Liang, & Zeger 2013). Unlike in their conditional counterpart, in a marginal framework the person effects are not modeled; they are integrated out. The interdependency between a person’s observations is then not implied by a random-effect structure but is explicitly modeled in a covariance matrix. As discussed below, if inferences about population-averages are the focus of research, a marginal approach greatly favors the parsimony of the model at hand and can offer several advantages in the context of parameter estimation and model selection.

A novel Bayesian Covariance Structure Model (BCSM) is proposed for clustered response times that is partly built on properties of a marginal modeling approach, but also explicitly accounts for the clustered structure of the data by modeling a structured covariance matrix. In the BCSM, the implied covariance structure of each random effect is separately modeled in the same additive covariance matrix, whereby each layer in the additive structure corresponds to one random effect. Therefore, the BCSM is a marginal modeling approach in which the dependence structure is explicitly modeled and hence preserved.

The BCSM differs from existing marginal modeling approaches, since the complete joint distribution of the observations is specified (and hence the complete likelihood of the model parameters). Thus, the BCSM preserves likelihood-based methods, which makes it possible to accommodate missing at random by default, the likelihoods usually give support to a unique maximum and can be used as the building blocks for a Bayesian modeling approach. This is not possible when using generalized estimating equations (GEE) to estimate a marginal model (Diggle et al., 2013; Liang & Zeger, 1986). In GEE, the covariance structure is treated as nuisance parameters and the focus lies solely on modeling the mean response. This avoids having to specify the conditional structure and therefore a possible misspecification of the same. A major downside of the GEE approach is that marginalization of different conditional structures can lead to inferentially identical models (Lee & Neider, 2004). This is the direct consequence of treating the covariance structures as nuisance parameters which do not have to be explicitly modeled to obtain consistent estimates. In other words, with an arbitrary covariance structure certain model assumptions cannot be checked for. Finally, contrary to the proposed framework, GEEs can be seen purely as an estimation procedure and do not allow common likelihood-based methods to assess the goodness-of-fit of a model, to compare models, to accommodate for missing at random, and to make inferences about model parameters.

To differentiate the proposed approach from existing marginal modeling methods, models constructed under the proposed framework are referred to as Bayesian Covariance Structure Models (BCSMs). BCSM offers three key advantages over a corresponding (conditional) random-effects model:

1. Tests for random-effect variances in mixed-effects models (e.g. Goldhammer & Kroehne, 2014) are complicated, as they require testing at the edge of the parameter space (Wood, 2013). These so-called boundary effects can lead to an underestimation of the statistical power of the corresponding tests and thus can bias the inferences made about the random-effect variance parameters of interest (Baguley, 2012, pp. 737–740). In a Bayesian framework, this problem is commonly tackled by choosing a more sophisticated prior distribution (e.g. Gelman, 2006; Gustafson, Hossain, & MacNab, 2006). The proposed BCSM, however, treats these parameters as covariances, which do not underlie the restriction of a lower or upper limit, as long as the positive definiteness of the covariance matrix is ensured. In line with that, boundary effects are reduced with truncated shifted inverse-gamma priors that allow the parameter space to cover negative values while enforcing sufficient rules for the positive definiteness of the covariance matrix. These priors are not as sharply peaked near zero as the default inverse-gamma priors and thus carry less information. Furthermore, in contrast to, for example, the half-Cauchy prior proposed by Gelman (2006), conjugacy is preserved. As a result, the hypothesis space is expanded to cover all likely parameter values and the availability of expressions of known forms for the conditional posterior distributions allows efficient Gibbs-sampling. In addition, given the proposed vague prior specification, more accurate estimates of very small random-effect variance parameters, respectively the corresponding covariances, can be obtained.
2. Specifying the effective number of parameters is trivial in the proposed framework, whereas in the random-effects model this forms an obstacle when applying model selection techniques such as the Bayesian Information Criterion (BIC) (Schwarz, 1978).
3. Estimation of random-effect variances is more likely to suffer from convergence issues with small sample sizes when compared to corresponding marginal models (Bell, John, & Jeffrey, 2008; Muth et al., 2016). This means that if the individual random effects themselves are not of interest and instead variance and covariance parameters are to be investigated, the proposed framework is of utility even when only limited data are available.

**Fig. 1:**
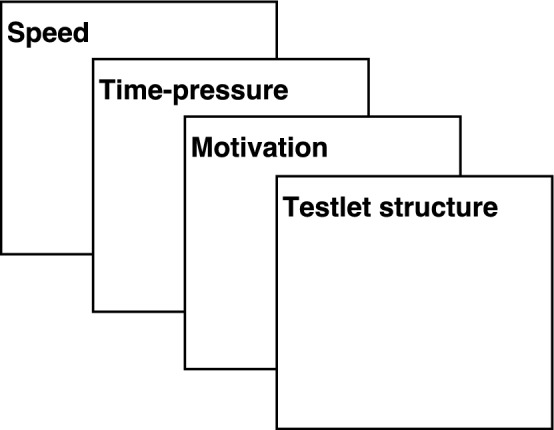
In an additive covariance structure, each explicitly modeled layer represents the influence of a random-effect variable on the interdependence between a person’s response times.

The BCSM for response times represents a multivariate generalization of the log-normal latent variable model (Klein Entink, Kuhn, Hornke, & Fox, 2009; van der Linden, 2006). A logarithmic transformation is applied to the naturally positively skewed distribution of response times, whereby the transformed response times of a person can be modeled with a normal distribution. In the conditional random-effect response time model, the observed response times are treated as realizations of a random variable and the corresponding probability distribution is determined by the items’ time intensity and the person’s speed. In the proposed BCSM for response times, the random effects themselves are not modeled. Instead, the implied interdependence between a person’s response times is modeled in an additive covariance structure. On the lowest level of the additive covariance structure, the interdependence between a person’s response times as implied by the person speed variable is modeled in a heterogeneous compound symmetric structure, where the measurement error variance parameters are free to vary across items. Therefore, in BCSM the random-effect variances are parameterized as covariance parameters. Latent variables such as time pressure, motivation, or the impact of testlet structures are not modeled but can cause local dependence within blocks of items. To take the additional sources of variation in a person’s response times into account, as illustrated by Fig. 1, the contribution of each latent variable on the interdependence of response times is explicitly modeled in its own layer in the additive covariance structure. This allows the estimation of the individual (co)variance parameters and makes it possible to evaluate hypotheses about the parameters. Therefore, a statement can be made about whether or not a certain latent variable or factor has an impact on the interdependence between a person’s response times (i.e., in the form of a test for local dependence within a block of items). As argued above, tests about the random-effect variances offer a more exhaustive hypothesis space and are satisfied with a smaller sample size when compared to a corresponding random-effects model. Finally, the random effects themselves are not modeled, but their values can be recovered from the model’s residuals.

The setup of the remaining text is as follows: a multivariate generalization of the log-normal response time model is specified within the BCSM framework. Extensions to include multidimensionality and factor loadings are discussed. Conjugate truncated shifted inverse-gamma priors are proposed that take into account the additive structure and positive definiteness of the covariance matrix, and resulting posteriors are derived. A Gibbs-sampling algorithm is defined with which samples from the full joint posterior can be obtained. A Bayes factor based on importance sampling and the BIC are discussed for the purpose of model selection in BCSM. Simulation studies are utilized to evaluate the proposed response time model’s performance in parameter recovery and model selection. The proposed response time model is applied to an empirical example in an educational measurement setting. Finally, the results, limitations, and future prospects of the BCSM framework are discussed.

## BCSM for Response Times

Before we define the response time model within the BCSM framework, we explain the notation as follows. The subscript *i* refers to the *i*-th person, *g* to the *g*-th group, and *k* to the *k*-th item. The number of persons in group *g* is denoted as $n_g$, and *N* stands for the total number of persons across all groups. Furthermore, the total number of groups and items is denoted as *G* and *p*, respectively. A bar over a data structure indicates the arithmetic mean over one or more dimensions that are specified by a dot in the subscript. For example, ${\bar{T}}_{.gk}$ denotes the mean log-response time over all persons in group *g* to item *k*. Finally, $\varvec{I}_p$ and $\varvec{J}_p$ are the identity matrix and a matrix of ones, each of dimension $p\times p$. The $p\times n_g$ data matrix $\varvec{T}_g$ contains the logarithmic transformation of the measured time that it took persons in group *g* to give a response to the respective items.

In the log-normal model for response times, the response times of a person are explained by a person parameter and an item parameter. The item parameter $\lambda _{gk}$ is the population-average log-response time for item *k* in group *g*. The person parameter $\zeta _{ig}$ represents the constant speed of person *i* in group *g* across all items and is assumed to follow a normal population distribution: $\zeta _{ig} \sim N(\mu _{\zeta _{g}}, \delta _{g})$. It thus expresses the deviation of the person’s speed from the population-average. This leads to the following equation for the log-response time of person *i* in group *g* to item *k*:

1 $$\begin{aligned} T_{igk}&= \lambda _{gk} - \zeta _{ig} + \varepsilon _{igk}, \, \varepsilon _{igk} \sim N(0, \sigma _{gk}^{2}). \end{aligned}$$

The person speed parameter $\zeta _{ig}$ in Eq. (1) can be replaced with the sum of the average population speed of group *g* ($\mu _{\zeta _{g}}$), and the error of the group’s population speed distribution $\varepsilon _{\zeta _{ig}}$:

2 $$\begin{aligned} T_{igk}&= \lambda _{gk} - (\mu _{\zeta _{g}} + \varepsilon _{\zeta _{ig}}) + \varepsilon _{igk} \nonumber \\&= \lambda _{gk} - \mu _{\zeta _{g}} + (\varepsilon _{\zeta _{ig}} + \varepsilon _{igk}) \nonumber \\&= \lambda _{gk} - \mu _{\zeta _{g}} + {\tilde{\varepsilon }}_{igk}. \end{aligned}$$

The error $\varepsilon _{igk}$ in the distribution of response times and the error of the population distribution of speed $\varepsilon _{\zeta _{ig}}$ are conditionally independent. From that, it follows that the sum of the error terms ${\tilde{\varepsilon }}_{igk}$ is normally distributed with a mean of zero and a variance of $\delta _{g} + \sigma _{gk}^{2}$. As illustrated by Eq. (3), due to the marginalization, the response times of a person to different items are correlated with the covariance parameter $\delta _{g}$. Given the above-mentioned marginalization, the covariance between the response times for two persons *i* and *j* of the same group *g* to items *k* and *l* is the following:

3 $$\begin{aligned} \hbox {Cov}(T_{igk}, T_{jgl})&= \hbox {Cov}(\lambda _{gk} - \mu _{\zeta _{g}} + \varepsilon _{\zeta _{ig}} + \varepsilon _{igk}, \lambda _{gl} - \mu _{\zeta _{g}} + \varepsilon _{\zeta _{jg}} + \varepsilon _{jgl}) \nonumber \\&= \hbox {Cov}(\varepsilon _{\zeta _{ig}} + \varepsilon _{igk}, \varepsilon _{\zeta _{jg}} + \varepsilon _{jgl}) \nonumber \\&= \hbox {Cov}(\varepsilon _{\zeta _{ig}}, \varepsilon _{\zeta _{jg}}) + \hbox {Cov}(\varepsilon _{igk}, \varepsilon _{jgl}) \nonumber \\&= {\left\{ \begin{array}{ll} \delta _{g} + \sigma _{gk}^{2} &{} \text {if } i = j, k = l \\ \delta _{g} &{} \text {if } i = j, k \ne l \\ 0 &{} \text {if } i \ne j \end{array}\right. }. \end{aligned}$$

Consequently, the response times of each person are multivariate log-normally distributed with a *p*-dimensional mean vector

4 $$\begin{aligned} \varvec{\mu }_{T_{g}} = [\lambda _{g1} - \mu _{\zeta _{g}}, \dots , \lambda _{gp} - \mu _{\zeta _{g}}] \end{aligned}$$

and the compound symmetry covariance matrix

5 $$\begin{aligned} \varvec{\Sigma }_{T_g}&= \hbox {diag}(\varvec{\sigma }_{g}^{2}) + \delta _g \varvec{J}_p \nonumber \\&= \begin{bmatrix} \delta _{g} + \sigma _{{g1}}^{2}&\delta _{g}&\dots&\delta _{g} \\ \delta _{g}&\delta _{g} + \sigma _{{g2}}^{2}&\dots&\delta _{g} \\ \vdots&\vdots&\ddots&\vdots \\ \delta _{g}&\delta _{g}&\dots&\delta _{g} + \sigma _{{gp}}^{2} \end{bmatrix}. \end{aligned}$$

Note that due to the marginalization, the mean and covariance structure is the same for all members of a group.

**Fig. 2:**
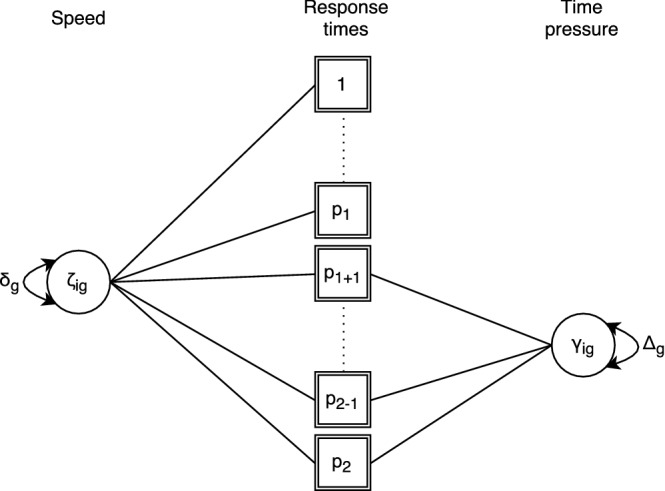
Multidimensionality in the interdependence between response times is realized through the additional covariance parameter $\Delta _g$. In a setting where the individual latent effects $\gamma _{ig}$ are not of interest for hypothesis testing and model selection, modeling the implied local dependence in the response time data is sufficient.

In the BCSM framework, the model specified in Eq. (5) describes the base layer of the additive covariance structure. Additional layers are modeled without modifying the mean structure specified in Eq. (4). As a result, multidimensionality in the interdependency of the response times can be introduced without including additional latent variables. Note that in the proposed model, each additional layer is explicitly modeled. This stands in contrast to an arbitrary covariance structure of a marginal model that is ambiguous about the corresponding conditional model. In the example illustrated by Fig. 2, persons are assumed to experience time pressure during the last part of the test. In a random-effects model, the time pressure effect would be represented by the latent variable $\gamma _{ig}$. That means that the variance of the random effects, i.e., $\hbox {Var}(\gamma _{ig}) = \Delta _g$, implies the dependence structure of a person’s response times. In the BCSM approach, only the dependence structure is modeled; $\gamma _{ig}$ itself is not modeled but would explain the specific dependence among response times to the affected (testlet) items of person *i* in the mean component. Note that $\Delta _g$ is parametrized as a covariance parameter in the BCSM. Furthermore, let $\varvec{u}_g$ be a *p*-dimensional design vector of 0’s and 1’s where a 1 indicates that the response times to an item are affected by $\gamma _{ig}$. Then, an additive covariance structure is obtained, which is a straightforward extension of Eq. (5):

6 $$\begin{aligned} \varvec{\Sigma }_{T_g}^{*}&= \varvec{\Sigma }_{T_g} + \Delta _g \varvec{u}_g\varvec{u}_g^T . \end{aligned}$$

Note that this extension is realized by modifying the covariance structure of the model with the addition of the covariance parameter $\Delta _g$. In other words, instead of modeling the individual effect of a time pressure ($\gamma _{ig}$) on a person’s response times, the implied covariance of a time pressure effect ($\Delta _g$) on the errors is modeled. Furthermore, note that no additional identification rules are required, as long as the design vectors are mutually distinct (i.e., no two $\varvec{u}_g$’s are the same). This holds for any pattern of an arbitrary number of additional layers.

BCSM is not limited to modeling the dependence structure implied by the single factor random intercepts model defined in Eq. (1). In fact, the described modeling approach generalizes to any covariance structure that can be expressed in the form of Eq. (6). This includes modeling the implied dependences of a random intercept and slope model [conditional: Eq. (7); BCSM: Eq. (8)], and of a testlet structure [conditional: Eq. (30); BCSM: Eqs. (31) and (32)]. Finally, dependences that are implied by correlated random effects are modeled directly in the additive covariance structure by specifying additional design vectors. Consequently, correlations between random effects are handled the same way as any other dependences in the data and do not require a modification of the described modeling approach.

**Table 1:** The dependences implied by correlated random effects are directly modeled in the additive covariance structure without modeling the random effects themselves.

Covariance layer	Design vector					
Speed	1	1	1	1	1	1
Testlet 1	1	1	0	0	0	0
Testlet 2	0	0	1	1	0	0
Testlet 3	0	0	0	0	1	1
Cross testlets 1, 2	1	1	1	1	0	0
Cross testlets 1, 3	1	1	0	0	1	1
Cross testlets 2, 3	0	0	1	1	1	1

This is realized through the specification of cross-covariances between testlets through additional design vectors. Each row corresponds to the design vector of one covariance layer.

**Fig. 3:**
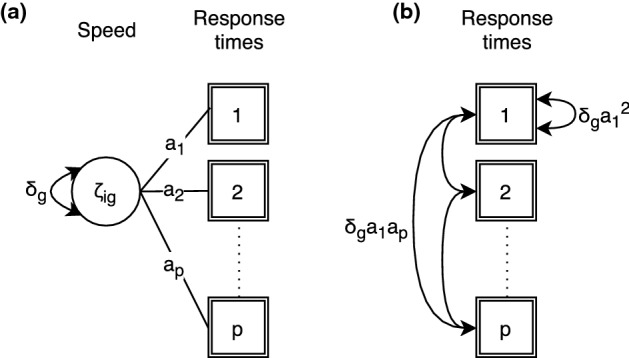
**a** In a random-effects model, time-discrimination parameters can be interpreted as item-specific factor loadings for the latent person speed variable. **b** In BCSM, the dependence structure implied by time-discrimination parameters is directly modeled without the inclusion of random effects. Measurement error variances are not shown.

As an illustration of modeling the dependence structure implied by correlated random effects, Table 1 contains the design vectors of a testlet RT BCSM for six items and three testlets. The first design vector specifies the dependences in the data that follow from the latent person speed variable. The next three rows specify the testlet structure, i.e., item 1 and 2, item 3 and 4, and item 5 and 6 each form a testlet. RTs to items in the same testlet are locally dependent. This dependence is explicitly modeled through the covariance parameter on the respective layer [i.e., $\Delta _g$ in Eq. (6)].

Following the same reasoning, the final three rows of Table 1 specify dependences between testlets. The corresponding covariance parameters can be interpreted as the covariances between testlet random effects in a random effects model. It is, however, important to note that BCSM is not limited to modeling dependences that are implied by random-effect structures. In particular, modeling negative interdependences (e.g., negative within-cluster correlations) poses a challenge in the random-effects modeling approach (e.g. El Leithy, Abdel Wahed, & Abdallah, 2016; Pryseley, Tchonlafi, Verbeke, & Molenberghs, 2011), but is straightforward and unambiguous in BCSM where dependences are modeled through covariance instead of variance parameters. Negative interdependences can furthermore naturally occur when jointly modeling different sorts of data, e.g., responses and response times (e.g. Klein Entink, Fox, & van der Linden, 2008; van der Linden, 2007).

Finally, factor loadings can be modeled in the proposed framework. An example is the time-discrimination parameter, which represents the quality of an item to discriminate between distributions of persons with a different level of speed (Klein Entink et al., 2008). The factor loading is included in the conditional response time model as an item-specific slope parameter $a_{gk}$:

7 $$\begin{aligned} T_{igk}&= \lambda _{gk} - a_{gk}\zeta _{ig} + \varepsilon _{igk}. \end{aligned}$$

Again, from this follows an additive covariance structure in the BCSM framework:

8 $$\begin{aligned} \varvec{\Sigma }_{T_g}&= \hbox {diag}(\varvec{\sigma }_{g}^{2}) + \delta _g\varvec{a}_{g}\varvec{a}_{g}^T. \end{aligned}$$

The corresponding random-effects model and its BCSM counterpart are shown in Fig. 3. Note that the resulting covariance matrix is not compound symmetric, but the properties necessary to build an additive structure are preserved. In fact, Eq. (8) removes the restriction of ${u}_{gk} \in \{0, 1\}$ in Eq. (6) and allows ${a}_{gk} \in {\mathbb {R}}$.

## Priors for Additive Covariance Matrices

In the proposed BCSM framework, the random-effect variance parameters are represented by covariance parameters. While covariance parameters do not underlie the restriction of being greater or equal to zero, to keep the covariance matrix positive definite certain lower bounds may not be crossed. The lower bounds are obtained through applying the Sherman–Morrison formula to the given problem (Lange, 2010, pp. 260–261) and are enforced by truncating the prior at hand.

A sufficient condition for the positive definiteness is defined for any additive layer *d* of a $p \times p$-dimensional covariance matrix $\varvec{A}$ of form

9 $$\begin{aligned} \varvec{A}_{d+1} = \varvec{A}_{d} + \psi \varvec{v}\varvec{v}^T, \end{aligned}$$

where $\psi$ is a scalar and $\varvec{v}$ is a vector of length *p*. From the Sherman–Morrison formula, it follows that

10 $$\begin{aligned} 1 + \psi \varvec{v}^T\varvec{A}_{d}^{-1}\varvec{v} > 0 \end{aligned}$$

is a sufficient condition for the positive definiteness of $\varvec{A}_{d+1}$, under the presumption that $\varvec{A}_{d}$ is also positive definite. The base layer $\varvec{A}_{1}$ follows a heterogenous compound symmetry structure:

11 $$\begin{aligned} \varvec{A}_{1}&= \hbox {diag}(\varvec{\sigma }^{2}) + \delta \varvec{1}_p\varvec{1}_p^T. \end{aligned}$$

From the condition defined in Eq. (10), it follows that $\min (\varvec{\sigma }^{2}) > 0$ and $\delta > -1/\varvec{1}_p^T \hbox {diag}(\varvec{\sigma }^{2})^{-1}\varvec{1}_p$ together ensure that $\varvec{A}_{1}$ is positive definite. If the base layer $\varvec{A}_{1}$ is positive definite, then the following condition is thus sufficient to ensure the positive definiteness of any additional layer:

12 $$\begin{aligned} \psi > -1/\varvec{v}^T \varvec{A}_{d}^{-1} \varvec{v} . \end{aligned}$$

Note that a closed-form expression for $\varvec{A}_{d}^{-1}$ can be derived from the Sherman–Morrison formula.

In line with the approach suggested by Fox, Mulder, and Sinharay (2017), shifted inverse-gamma priors are defined for the variance and covariance parameters. To ensure the positive definiteness of the covariance matrix, the condition defined in Eq. (12) is implemented through the indicator function $\mathbb {1}_t$. From this follows an extended inverse-gamma distribution with four parameters, where $\upsilon$ is the shift parameter and $\tau$ is the truncation point:

13 $$\begin{aligned} IG(x, \alpha , \beta , \upsilon , \tau ) = \left[ \frac{\beta ^{\alpha }}{\Gamma (\alpha )} (x + \upsilon )^{-\alpha -1} \exp \left( -\frac{\beta }{x + \upsilon }\right) \right] \cdot \mathbb {1}_t(x > \tau ). \end{aligned}$$

Note that $\tau = -\upsilon$ equals an untruncated shifted inverse-gamma distribution and $\tau = \upsilon = 0$ equals a default inverse-gamma distribution.

Consequently, the priors for the covariance and variance parameters can be written as

14 $$\begin{aligned} \pi (\delta _g|\varvec{\sigma }_{g}^{2})&= IG(\delta _g, \alpha _0, \beta _0, {\bar{\sigma }}_g^{2}/p, -1/\varvec{1}_p^T \hbox {diag}(\varvec{\sigma }_{g}^{2})^{-1}\varvec{1}_p) \end{aligned}$$

and

15 $$\begin{aligned} \pi (\varvec{\sigma }_{g}^{2}|\delta _g)&= \prod _{k=1}^p IG(\sigma _{gk}^2, \alpha _0, \beta _0, \delta _g, 0). \end{aligned}$$

For covariance parameters in additional layers, the truncation point changes according to Eq. (12). Note that the priors are defined in a conditional form, e.g., $\pi (\delta _g|\varvec{\sigma }_{g}^{2})$ and $\pi (\varvec{\sigma }_{g}^{2}|\delta _g)$. This is sufficient for the Markov chain Monte Carlo (MCMC) algorithm. For Bayes factor testing, the joint prior, e.g., $\pi (\delta _g, \varvec{\sigma }_{g}^{2})$, can be constructed as the product of the (estimated) marginal priors.

## Posterior Distributions

Given Eq. (5), the covariance between two responses times of a person *i* in group *g* for the *k*-th and/or *l*-th item is the following:

16 $$\begin{aligned} \hbox {Cov}(T_{igk}, T_{igl}) = \delta _g + \sigma _{gk}^2 \cdot \mathbb {1}\left( k = l\right) , \end{aligned}$$

where $\mathbb {1}$ is the indicator function. Note that the total variance of a person’s response time consists of a between-subject part ($\delta _g$) and a within-subject part ($\sigma _{gk}^2$). The terms between-subject and within-subject follow from the assumption that all persons within a group share a common covariance structure.

The between sum of squares

17 $$\begin{aligned} \hbox {SSB} = \sum _{i=1}^{n_g} \left( {\bar{T}}_{ig.} - {\bar{T}}_{.g.} \right) ^2, \end{aligned}$$

is a sufficient statistic for the covariance parameter $\delta _g$. In fact, multiplying the likelihood of the person means

18 $$\begin{aligned} p({\bar{T}}_{1g.} \ldots {\bar{T}}_{ng.} | {\bar{\sigma }}_g^2, \delta _g)&= (2p\pi )^{-\frac{n_g}{2}}({\bar{\sigma }}_g^{2}/p + \delta _g)^{-\frac{n_g}{2}} \exp \left( - \frac{\mathrm{SSB}/2}{{\bar{\sigma }}_g^{2}/p + \delta _g}\right) , \end{aligned}$$

with the conjugate truncated shifted inverse-gamma prior specified in Eq. (14) leads to the conditional posterior of $\delta _g$:

19 $$\begin{aligned}&p(\delta _g | {\bar{T}}_{1g.} \ldots {\bar{T}}_{ng.} {\bar{\sigma }}_g^{2})\nonumber \\&\quad = \left[ \frac{(\beta _0 + \mathrm{SSB}/2)^{(\alpha _0 + n_g/2)}}{\Gamma (\alpha _0 + n_g/2)} (\delta _g + {\bar{\sigma }}_g^{2}/p)^{-(\alpha _0 + n_g/2)-1} \exp \left( -\frac{\beta _0 + \mathrm{SSB}/2}{\delta _g + {\bar{\sigma }}_g^{2}/p}\right) \right] \nonumber \\&\qquad \cdot \mathbb {1}_t(\delta _g > -1/\varvec{1}_p^T \hbox {diag}(\varvec{\sigma }_{g}^{2})^{-1}\varvec{1}_p). \end{aligned}$$

Similarly, the within sum of squares of component *k*, $\hbox {SSW}_k = \sum _{i=1}^{n_g}(T_{igk} - {\bar{T}}_{.gk})^2$, is a sufficient statistic for the corresponding measurement error variance parameter. Given the prior specified in Eq. (15), the posterior is a truncated shifted inverse-gamma distribution with shift parameter $\delta _g$ and a truncation that ensures that $\sigma _{gk}^{2} > 0$:

20 $$\begin{aligned} \sigma _{gk}^{2} \sim IG(\alpha _0 + n_g/2, \beta _0 + \hbox {SSW}_k/2, \delta _g, 0). \end{aligned}$$

To ease the derivation of Bayes factors about the invariance of measurement error variance parameters within or across groups, it is useful to sample the mean variance ${\bar{\sigma }}_g^{2}$ directly as an auxiliary parameter. As proved in “Appendix B”, the posterior is also truncated shifted inverse-gamma:

21 $$\begin{aligned} {\bar{\sigma }}_g^{2} \sim IG(\alpha _0 + n_g/2, \beta _0 + \hbox {SSW}/(2p), \delta _g, 0), \end{aligned}$$

where $$\hbox {SSW} = \sum _{i=1}^{n_g} \sum _{k=1}^{p} \left( T_{igk} - {\bar{T}}_{.gk} \right) ^2$$.

Like the covariance parameter $\delta _g$ in the base layer, the posterior of a covariance parameter $\Delta _{gd}$ in any additional layer *d* is shifted inverse-gamma distributed with a truncation to ensure the positive definiteness of the resulting covariance matrix. For example, if $d = 2$,

22 $$\begin{aligned} \Delta _{g2} \sim IG(\alpha _0 + n_g/2, \beta _0 + \mathrm{SSB}_{\Delta _{g2}}/2, {\bar{\sigma }}_{g2}^{2}/p_2 + \delta _g, t_{\mathrm{PSD}_{\Delta _{g2}}}), \end{aligned}$$

where ${\bar{\sigma }}_{g2}^{2}/p_2$ is the average measurement error variance across the items that are affected by the additional covariance layer (i.e., items selected in the corresponding design vector $\varvec{u}_{\Delta _{g2}}$ divided by the number of affected items $p_2$). Furthermore,

$$\begin{aligned} \mathrm{SSB}_{\Delta _{g2}}/2 = \sum _{i=1}^{n_g} \left( \frac{1}{p_2} \sum _{k \in \varvec{u}_{\Delta _{g2}}}^{p_2}({T}_{igk}) - \frac{1}{n_g p_2} \sum _{i=1}^{n_g}\sum _{k \in \varvec{u}_{\Delta _{g2}}}^{p_2}({T}_{igk}) \right) ^2 \end{aligned}$$

and $t_{\mathrm{PSD}_{\Delta _{g2}}}$ is the truncation point following from Eq. (12). Note that Eq. (22) can be generalized to any number of additive layers by recursively computing the shift parameter and truncation point based on the layers below the current layer in the resulting covariance matrix.

## Bayesian Inference

A Gibbs-sampling algorithm is specified with which samples from the full joint posterior distribution of the BCSM for response times can be drawn. As outlined in Algorithm 1, after the initialization phase, the item parameters, group parameters, measurement error variance parameters, and covariance parameters are sampled iteratively from their respective conditional posterior distribution. Finally, posterior mean estimates of the respective parameters are computed as the arithmetic mean of the MCMC samples while taking a burn-in phase into account.

To identify the model, the mean of the item parameters is assumed to be equal across groups; that is, ${\bar{\lambda }}_g = {\bar{\lambda }}_h$ for groups *g* and *h*. Furthermore, the group speed mean is fixed to zero in the first group ($\mu _{\zeta _{1}} = 0$). This rescaling is done via the (posterior) MCMC samples. Thereby, a distinction is made between the (untransformed) freely estimated parameters, for which a prior is specified, and the constrained (rescaled) parameters that are used for further computations (e.g. Fox, Klein Entink, & van der Linden, 2007; Luo & Jiao, 2018). For the fixed item and group effects, a locally uniform prior is defined. Finally, data missing at random $\varvec{\omega }_g$ is properly imputed by drawing samples from the posterior predictive distribution of the data in each iteration. See “Appendix A” for details on the sampling steps.
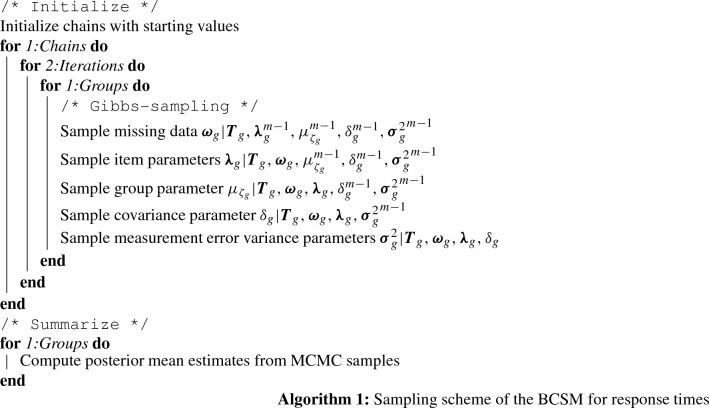


## Bayes Factor Testing

A Bayes factor quantifies the relative evidence of two competing models. More specifically, it is the ratio of evidence for each model times the a priori assumptions about the evidence, that is, the prior odds (Kass & Raftery, 1995):

23 $$\begin{aligned} \hbox {BF}_{01}&= \frac{m\left( \varvec{T}; M_0\right) }{m\left( \varvec{T}; M_1\right) } \cdot \frac{\pi _0}{\pi _1}. \end{aligned}$$

Being a priori by nature, the prior odds $\frac{\pi _0}{\pi _1}$ incorporate information such as former research results or expert opinions and are not derived in the process of computing the Bayes factor. Thus, Eq. (23) simplifies to a ratio of marginal likelihoods. The marginal likelihood of the data under a model $M_b$ is obtained by integrating the probability density function of the data with respect to the prior density:

24 $$\begin{aligned} m\left( \varvec{T}|M_b\right) = \idotsint p(\varvec{T}|\phi _1, \ldots , \phi _z, M_b)\pi (\phi _1, \ldots , \phi _z|M_b) \,\mathrm{d}\phi _1 \ldots \mathrm{d}\phi _z, \end{aligned}$$

where $\phi _1, \ldots , \phi _z$ are the model parameters of interest for the given Bayes factor. An estimator for the marginal likelihood is constructed based on the importance sampling technique proposed by Perrakis, Ntzoufras, and Tsionas (2014). In importance sampling, instead of integrating with respect to the prior density as in Eq. (24), the integration is applied with respect to an importance sampling density $g(\phi _1, \ldots , \phi _z|M_b)$. As illustrated by Perrakis et al. (2014), using the product of the marginal posterior distributions of the parameters of interest as the importance sampling density, that is, $g(\phi _1, \ldots , \phi _z|\varvec{T}, M_b) = \prod \nolimits _{u=1}^{z} p(\phi _u|\varvec{T}, M_b)$, leads to an estimator with desirable properties: first, it is unbiased; second, it has a finite variance; and third, it handles any unknown constants in the prior distributions as long as the corresponding marginal posteriors are included in the importance sampling density. The resulting integral

25 $$\begin{aligned} m\left( \varvec{T}|M_b\right)&= \idotsint \frac{p\left( \varvec{T}|\phi _1, \ldots , \phi _z, M_b\right) \pi \left( \phi _1, \ldots , \phi _z|M_b\right) }{\prod \nolimits _{u=1}^{z} p\left( \phi _u|\varvec{T}, M_b\right) } \prod \limits _{u=1}^{z} p\left( \phi _u|\varvec{T}, M_b\right) \mathrm{d}\phi _u \end{aligned}$$

is estimated by

26 $$\begin{aligned} {\hat{m}}\left( \varvec{T}|M_b\right)&= \frac{1}{J} \sum _{j=1}^{J} \frac{p\left( \varvec{T}|\phi _1^{(j)}, \ldots , \phi _z^{(j)}, M_b\right) \pi \left( \phi _1^{(j)}, \ldots , \phi _z^{(j)}|M_b\right) }{\prod \nolimits _{u=1}^{z} p\left( \phi _u^{(j)}|\varvec{T}, M_b\right) }, \end{aligned}$$

where $\phi _1^{(j)}, \ldots , \phi _z^{(j)}$ are draws from the respective marginal posterior distributions and *J* is the number of MCMC samples utilized to estimate the marginal likelihood. Draws from the marginal posterior distributions are obtained by permuting the samples from the full joint posterior distribution (Perrakis et al., 2014, pp. 5–6): before randomly reordering each column (corresponding to the posterior sample of one model parameter) of the MCMC chain, the draws within each row (corresponding to one MCMC iteration) are naturally correlated draws from the conditional posterior distributions. After re-ordering, each row represents decorrelated draws from the marginal posterior distributions. The marginal posterior probabilities in the denominator and the marginal prior probabilities in the numerator of Eq. (26) are estimated through Rao-Blackwellization (Gelfand & Smith, 1990). In the case of data missing at random, the missing data parameters $\varvec{\omega }$ do not provide additional information about the model evidence. Therefore, the marginal likelihood estimation is based solely on the observed data.

A straightforward example of the estimator specified in Eq. (26) is the evaluation of evidence in favor of the hypothesis that the covariance parameter is unrestricted ($H_1: \delta \ne 0$) against evidence supporting the complementary hypothesis ($H_2: \delta = 0$):

27 $$\begin{aligned} \hbox {BF}_{12}&= \frac{ \frac{1}{J} \sum _{j=1}^{J} \frac{p\left( \varvec{T}|\delta ^{(j)}, \varvec{\sigma }^{2(j)}, \varvec{\xi }^{(j)}, M_1\right) \pi \left( \delta ^{(j)}, \varvec{\sigma }^{2(j)}, \varvec{\xi }^{(j)}|M_1\right) }{p\left( \delta ^{(j)}|\varvec{T}, M_1\right) p\left( \varvec{\sigma }^{2(j)}|\varvec{T}, M_1\right) p\left( \varvec{\xi }^{(j)}|\varvec{T}, M_1\right) } }{ \frac{1}{J} \sum _{j=1}^{J} \frac{p\left( \varvec{T}|\delta ^{(j)}, \varvec{\sigma }^{2(j)},\varvec{\xi }^{(j)}, M_2\right) \pi \left( \varvec{\sigma }^{2(j)}, \varvec{\xi }^{(j)}|M_2\right) }{p\left( \varvec{\sigma }^{2(j)}|\varvec{T}, M_2\right) p\left( \varvec{\xi }^{(j)}|\varvec{T}, M_2\right) } }, \end{aligned}$$

where $\pi (\delta | M_2)$ has a point mass at $\delta = 0$, and $\varvec{\xi }$ is a block of nuisance parameters (e.g., item and group intercepts). If necessary, multiple blocks of nuisance parameters can be specified. Note that possible unknown normalizing constants of the priors also appear in the corresponding marginal posterior densities, thus canceling out. The estimator specified in Eq. (26) is furthermore suited to obtain evidence under order-restricted hypotheses such as $H3: \delta _1 < \delta _2$, in which case the parameter space is constrained in some way (e.g. Gelfand, Smith, & Lee, 1992; Klugkist, Laudy, & Hoijtink 2005; Wagenmakers, Lodewyckx, Kuriyal, & Grasman, 2010).

In a setting where priors are deemed to be purely objective, an efficient approach to obtaining an estimate of the marginal likelihood of the data is the Laplace approximation (Bruijn, 1970, Chapter 4). Therefore, in this situation an appropriate method for comparing model evidence is the BIC:

28 $$\begin{aligned} \hbox {BIC}_m&= -2\log ({\hat{L}}_m) + d_mlog({\hat{N}}^{*}), \end{aligned}$$

where *m* refers to the *m*-th model, ${\hat{L}}_m$ is the likelihood of the data given the posterior mean estimates of the model parameters, $d_m$ is the number of free parameters under the model, and ${\hat{N}}^{*}$ is the sample size. Note that in BCSM, random effects are not estimated. Therefore, compared to linear mixed-effects models, shrinkage is greatly reduced in BCSMs and a better approximation of the effective number of parameters is achieved. Under a vague prior specification (i.e., small shape and large scale parameters), asymptotically, the effective number of parameters in the BCSM is equal to the number of fixed effects plus the parameters in the covariance matrix (e.g. Overholser & Xu, 2014). A safe choice for the effective sample size is the total number of observations ${\hat{N}}^{*} = pN$ (Faes, Molenberghs, Aerts, Verbeke, & Kenward, 2009). A Bayes factor for two competing models 0 and 1 can be approximated given the respective BICs:

29 $$\begin{aligned} \hbox {BF}_{01}&= \frac{m\left( \varvec{T}; M_0\right) }{m\left( \varvec{T}; M_1\right) } \approx \exp \left( \frac{- \Delta \mathrm{BIC}_{01}}{2} \right) , \end{aligned}$$

where $$\Delta \hbox {BIC}_{01} = \hbox {BIC}_0 - \hbox {BIC}_1$$.

## Simulation Studies

Two simulation studies are conducted. The first simulation study aims at evaluating the estimation of testlet (co)variance parameters close to zero and the coverage rate of the relevant credible intervals. In that context, a BCSM testlet model for response times is compared to a random-effect testlet model. In the second simulation study, a Bayes factor for the local independence of response times within testlets is evaluated under different sample sizes and population values of the testlet (co)variance parameters. Both simulation studies are based on a test that consists of testlets: blocks of items that relate to a common content area (Wainer & Kiely 1987).

A testlet structure implies that a person’s response times can be more alike within a testlet than across testlets. In other words, the grouping of similar items introduces dependence between a person’s response times within a testlet. This dependence is not accounted for by merely controlling the persons’ constant working speed across the test. Consequently, random-effects models commonly introduce a person–testlet interaction effect into the model equation (e.g. Hecht, Siegle, & Weirich, 2017; Wang & Wilson, 2005):

30 $$\begin{aligned} T_{igk} = \lambda _{gk} - (\zeta _{ig} + \theta _{igj(k)}) + \varepsilon _{igk}, \, \varepsilon _{igk} \sim N(0, \sigma _{gk}^{2}), \end{aligned}$$

where *j*(*k*) denotes an item *k* in testlet *j* and $\theta _{igj(k)}$ is the corresponding person–testlet interaction effect. The random speed and person–testlet interaction effects are normally distributed, with $\zeta _{ig} \sim N(\mu _{\zeta _{g}}, \delta _g)$ and $\theta _{igj(k)} \sim N(\mu _{\theta _{gj}}, \Delta _{gj})$. To identify the model, the variance of the random speed effects is fixed. In the BCSM approach, the person–testlet interaction effects are not modeled in the mean term; that is,

31 $$\begin{aligned} \varvec{T}_{ig} = \varvec{\lambda }_{g} - (\mu _{\zeta _{g}} + \varvec{\mu }_{\theta _{g(k)}}) + \varvec{\varepsilon }_{ig}, \, \varvec{\varepsilon }_{ig} \sim N(\varvec{0}_{p}, \varvec{\Sigma }_{g}). \end{aligned}$$

Instead, the additive covariance structure is extended with an additional layer and covariance parameter for each testlet. For a test consisting of $N_t$ testlets, this results in an additive covariance structure with $N_t + 1$ layers: The first layer follows from the influence of the latent speed on the response times, and the remaining layers represent the contribution of each testlet effect on the dependence of a person’s response times. Therefore, like the person speed parameters, the person–testlet interaction effects themselves are not modeled. Instead, the dependence between a person’s response times within a testlet is explicitly modeled in the covariance structure of the error term. The part of the dependence between response times that is assumed to be explained by the latent person speed is operationalized as the covariance parameter $\delta _g$. The part of the dependence between response times that is assumed to be explained by the testlet structure, while keeping the latent speed constant, is operationalized as $\Delta _{gj}$. The additive layer structure is represented by the following covariance matrix:

32 $$\begin{aligned} \varvec{\Sigma }_{g}&= \hbox {diag}(\varvec{\sigma }_{g}^{2}) + \delta _g \varvec{J}_p + \sum _{j=1}^{N_t} \Delta _{gj} \varvec{u}_{gj}\varvec{u}_{gj}^T, \end{aligned}$$

where $\varvec{u}_{gj}$ is a *p*-dimensional design vector specifying which items belong to testlet *j* in group *g*.

### Parameter Estimation and Credible Intervals

To measure the precision and bias of testlet (co)variance parameter estimates and the coverage rate of the corresponding credible intervals a simulation experiment is conducted. The number of test-takers ($N = 300$), the length of the test ($p = 30$), and the number of testlets ($N_t = 3$) are fixed across the 1000 replications. All test-takers are part of the same group. The population values of the first three testlet (co)variance parameters are $\Delta _{g1} = 0$, $\Delta _{g2} = .01$ and $\Delta _{g3} = .05$. The remaining population parameters are $\delta _g = .2$, $\mu _{\zeta _{g}} = \mu _{jg} = 0$, $\lambda _{gk}\sim N(0, 1)$ and $\sigma _{gk}^{2} = \varvec{1}_p$. Data are generated under the restrictions of the respective models: (a) the a priori assumption about whether or not the testlet (co)variance parameters may be negative is taken into account when simulating response times; and (b) to identify the random-effects model, the variance of the random speed effects is fixed, that is, $\zeta _{ig}\sim N(0, .2)$. Consequently, the variance is also fixed when generating data for the random-effects models. Note that all parameters in the BCSM covariance structure are free, i.e., $\delta _g$ is not fixed for the BCSM.

Both the BCSM and the random-effects model are fitted in a Bayesian framework. The Gibbs-sampling algorithm of the BCSM framework is implemented in R (R Core Team, 2017), and the random-effects model is fitted with the R package R2jags (Su, 2015). Estimates refer to the mean of the respective posterior distributions and the credible intervals are equally-tailed. The coverage rate of the credible intervals and the distribution of the posterior mean estimates is based on 1000 replications. Each replication consists of 10,000 MCMC iterations, and a burn-in phase of 10% is applied. For the BCSM, a truncated shifted inverse-gamma prior with $\hbox {shape} = 10^{-8}$ and $\hbox {scale} = 10^{8}$ is defined for the testlet (co)variance parameters. For the random-effects model, a default non-informative inverse-gamma prior on the variance parameters is likely to cause the MCMC sample chains of very small variance parameters to often get stuck at zero (Browne, Steele, Golalizadeh, & Green, 2009; Lesaffre & Lawson, 2012). In practice, the resulting autocorrelation renders obtaining information about the posteriors given a reasonable number of MCMC iterations futile. As a remedy, parameter expansion is implemented through a half-Cauchy prior with $\hbox {mode} = 0$ and $\hbox {scale} = 25$ on the testlet standard deviation parameters as proposed by Gelman (2006).

A visual inspection of the model parameters’ trace plots showed no evidence against convergence of the MCMC algorithms. The results of the parameter estimation and the coverage rates are shown in Table 2. Due to the skewness of the respective distributions, the posterior mean estimates of the two smallest testlet variance parameters are positively biased for the random-effects model. Under the BCSM, no bias is observed. The standard deviation of the posterior mean estimates is smaller for the random-effects model. However, as described above, fewer restrictions were applied when generating data for the BCSM. Under the BCSM, the empirical coverage rates correspond to the theoretical coverage of the credible intervals. Under the random-effects model, a true value of zero is not included in any of the computed 95%-credible intervals. For true values close to zero, a significant undercoverage is observed.

**Table 2:** Upper part: mean and standard deviation of posterior mean estimates of testlet (co)variance parameters. Lower part: empirical coverage of corresponding 95%-credible intervals.

	Trunc. shifted IG	Half-Cauchy
$\Delta$	*Empirical mean (SD) of posterior mean estimates*	
0	.000 (.034)	.021 (.015)
.01	.011 (.033)	.026 (.019)
.05	.051 (.037)	.054 (.030)

$\Delta$	*Empirical coverage of 95%-credible intervals*	
0	94.3	0
.01	95.1	84.5
.05	95.5	75.4

Results based on 1000 simulated replications with $N = 300$ persons, $p = 30$ items and $N_t$ = 3 testlets.

### Model Selection

**Fig. 4:**
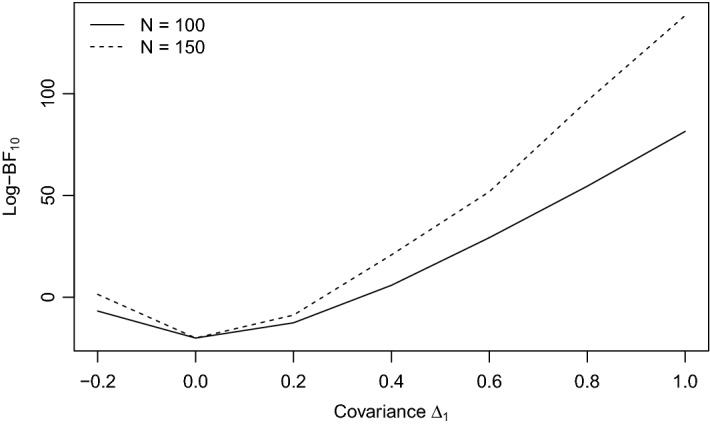
Average log-Bayes factor across 50 replications quantifying the evidence for $H_a: \Delta _1 \ne 0$ against the evidence for $H_0: \Delta _1 = 0$. A positive value indicates that $H_a$ is more plausible. The comparison is made for three groups of size $N_1 = N_2 = N_3 = 100$, respectively $N_1 = N_2 = N_3 = 150$.

Given a testlet structure, the assumption of local independence states that a person’s response times within and between testlets are independent when controlling for the person’s speed. In the random-effects model specified in Eq. (30) and the proposed BCSM specified in Eq. (32), the assumption of local independence is violated if the testlet (co)variance is not equal to zero. In this simulation, the plausibility of two versions of the model described in Eq. (32) is evaluated with a Bayes factor. In the null model $M_0$, the covariance parameter $\Delta _1$ is restricted to zero. In the alternative model $M_a$, all covariance parameters are unrestricted. In other words, according to the null model local independence holds for the items within the first testlet, and the alternative hypothesis indicates local dependence. The model evidence is compared between $M_0$ and $M_a$ for a set of 7 population values of $\Delta _1$, namely $\{-\,.2, 0, .2, .4, .6, .8, 1\}$. For each value of $\Delta _1$, 50 samples are drawn from the respective population and for each sample the log-Bayes factor is computed. This is done twice, first for three groups of test-takers of size $N_1 = N_2 = N_3 = 100$ and second for a group size of $N_1 = N_2 = N_3 = 150$. In both cases, data for $p = 18$ items are simulated and 3000 MCMC iterations are run. A truncated shifted inverse-gamma prior with $\hbox {shape} = 10^{-3}$ and $\hbox {scale} = 10^{3}$ is defined for the testlet covariance parameters. The means of each replication are summarized in Fig. 4.

**Fig. 5:**
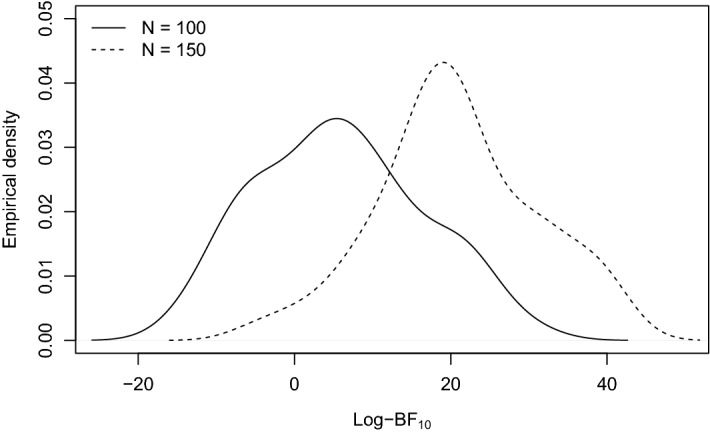
Empirical density of the log-Bayes factor across 50 replications quantifying the evidence for $H_a: \Delta _1 \ne 0$ against the evidence for $H_0: \Delta _1 = 0$. A positive value indicates that $H_a$ is more plausible. Samples are drawn from a population with $\Delta _1 = .4$. The comparison is made for three groups of size $N_1 = N_2 = N_3 = 100$, respectively $N_1 = N_2 = N_3 = 150$.

The Bayes factor behaves as expected: a larger discrepancy between the population value of $\Delta _1$ and zero makes the alternative model more plausible. Figure 5 shows the empirical density of the log-Bayes factor at $\Delta _1 = .4$ for group sizes of $N = 100$ and $N = 150$. Both figures illustrate that more data lead to a greater statistical power.

## Empirical Example

Realistic Mathematics Education (RME) is an approach to teaching and learning theory that is based on the idea of providing students with problems that are perceived as useful and relevant. This aims at making mathematical education accessible to a wider range of students and therefore giving numeracy a stronger focus in society. Based on an empirical dataset by Buschers (2016), we investigated whether or not presenting contextual numeracy items in different formats has an effect on students’ response processes. To gain insight into the latent response processes, response times are utilized (Molenaar, Tuerlinckx, & Maas, 2014). Three different presentation formats were considered: text only, image only and text and image (see Table 3 for an example). In general, a numeracy problem in a test should not overload or distract the student with redundant information and should furthermore ensure that the available information is easily accessible to the student. For the given empirical example, the focus is on the translation of the contextual problem to a mathematical problem. In this context, two cognitive theories are considered. First of all, the cognitive load theory states that the cognitive capacity of a student, and in particular his or her short term memory, is limited and can thus be overloaded. Hence, in the majority of cases it seems better to not repeat the information included in an image in the accompanying text. Second, according to the dual channel principle students have separate channels to process verbal and pictorial information. Therefore, it is not only the amount of information that plays a role, but also how the information is presented and thus processed. For example, it can be argued that presenting information in text and image allows the student to focus on the format of presentation, or combination thereof, that works best for him or her.

In total, data from 301 respondents were recorded in various Dutch schools. The randomly assigned groups are of size $N_1 = 99$, $N_2 = 96$ and $N_3 = 94$ when including respondents for whom data for at least five items were available. The respondents are students of the three levels of prevocational education and the third level of vocational education. A partially counterbalanced Latin square design with three blocks is employed. Each block corresponds to one of the three presentation formats. Thus, within the three randomly assigned groups, each student is presented all $p = 35$ items, but with different formats. The order of the presentation format varies per group in accordance with the Latin square design. Within each group, item order effects are possible. These effects are accounted for when the three counterbalanced groups are merged for the purpose of statistical inference (i.e., parameter estimation and hypothesis testing).

The goal of this research is to investigate whether or not the students’ performance differs between the three item presentation formats. This is operationalized as differences in response times between the three variants. More specifically, it is of interest whether or not the response times within one variant are more alike than the response times across all variants. Furthermore, if it is plausible that the response times within two variants are more alike than the response times across all variants, then comparing the covariance within the two variants is of interest. The plausibility of the hypotheses is evaluated with the approximated Bayes factor specified in Eq. (29).

**Table 3:**
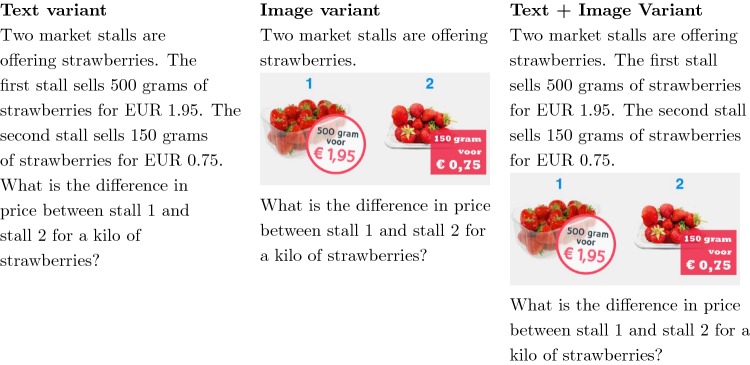
*Note.* Reprinted from Words, pictures or both?: the influence of the presentation of contextual numeracy problems on student performance in (pre) vocational education, by Buschers (2016), *unpublished Master’s thesis*, p. 7.

### The Statistical Model

Each item presentation format variant corresponds to a separate testlet *j* in a testlet structure. The item–testlet combination (i.e., which item belongs to which testlet) varies across the three groups of students. An appropriate mixed-effects model is the following:

33 $$\begin{aligned} T_{igk}&= \lambda _{gk} - (\zeta _{ig} + \theta _{igj(k)}) + \varepsilon _{igk}, \, \varepsilon _{igk} \sim N(0, \sigma _{gk}^{2}), \end{aligned}$$

where the random speed and testlet effects are normally distributed with $\zeta _{ig} \sim N(\mu _{\zeta _{g}}, \delta _g)$ and $\theta _{igj(k)} \sim N(\mu _{\theta _{gj}}, \Delta _{gj})$. The goal of the present research is to make inferences about the effect of the testlet structure (i.e., the different item presentation formats) on the interdependence between a person’s response times. More specifically, it is of interest to evaluate whether or not response times are more alike within a testlet than across testlets. This translates into a statement about the local dependence of a person’s response times within each testlet. For the given application, the focus thus lies on the population-average of the (co)variance parameters $\Delta _{gj}$ and not on the individual random effects, i.e., $\theta _{igj(k)}$.

In the BCSM framework, the mixed-effects model in Eq. (33) is marginalized and the interdependency between a person’s response times is modeled in an additive covariance matrix with four layers:

34 $$\begin{aligned} \varvec{\Sigma }_{T_g}&= \left[ \hbox {diag}(\varvec{\sigma }_{g}^{2}) + \delta _g \varvec{J}_p\right] + \left[ \Delta _{g1} \varvec{u}_{g1}\varvec{u}_{g1}^T\right] + \left[ \Delta _{g2} \varvec{u}_{g2}\varvec{u}_{g2}^T\right] + \left[ \Delta _{g3} \varvec{u}_{g3}\varvec{u}_{g3}^T\right] , \end{aligned}$$

where $\delta _g$ describes the covariance across all items and $\Delta _{g1}$, $\Delta _{g2}$ and $\Delta _{g3}$ describe the additional covariance within the variants “Text”, “Image” and “Text and Image”. The fact that the testlet effect $\theta _{igj(k)}$ has three categories is represented in the BCSM by the design vectors $\varvec{u}_{g1}$, $\varvec{u}_{g2}$ and $\varvec{u}_{g3}$, which thus specify the order of the presentation formats in group *g*. Two rules are defined to identify the model. First, the group speed mean is set to zero in all groups ($\mu _{\zeta _{1}} = \mu _{\zeta _{2}} = \mu _{\zeta _{3}} = 0$). As a result, the time intensity parameters are on the same scale across groups, which allows the extraction of the presentation variant effects. Second, the measurement error variance parameter of the last item ($\sigma _{p}^{2}$) is set to be equal in all groups. This ensures that the covariance parameters are on the same scale across groups (e.g., $\Delta _{11} = \Delta _{21} = \Delta _{31}$). A truncated shifted inverse-gamma prior with $\hbox {shape} = 10^{-3}$ and $\hbox {scale} = 10^{3}$ is defined for the variance and covariance parameters. For the fixed item effects ($\lambda _{gk}$) a locally uniform prior is approximated with $N(0, 10^{10})$. Finally, data are assumed to be missing at random (MAR).

### Results

The model parameters are estimated with one MCMC chain of 50,000 iterations. A burn-in phase of 10% is applied. A visual inspection of the model parameters’ trace plots showed no evidence against convergence of the MCMC algorithm. The posterior mean estimate of the covariance parameter across all variants is .417 (SD .135). The additional covariances in the “Text”, “Image” and “Text and Image” variants are estimated as .100 (SD .130), .036 (SD .071) and .043 (SD .082), respectively. The plausibility of the hypothesis stating that response times are more alike within a variant than across variants is evaluated with an approximated Bayes factor by comparing the evidence for said hypothesis to the evidence in favor of the respective complementary null hypotheses: $H_{01}: \Delta _{1} = 0$, $H_{02}: \Delta _{2} = 0$ and $H_{03}: \Delta _{3} = 0$.

**Table 4:** Approximated log-Bayes factor quantifying the plausibility of the alternative hypothesis, i.e., response times within a presentation format variant are more alike than response times across variants, against the null hypothesis; i.e., the response times are not more alike.

	$H_{a1}: \Delta _{1} \ne 0$	$H_{a2}: \Delta _{2} \ne 0$	$H_{a3}: \Delta _{3} \ne 0$
$\hbox {Log-BF}_{\mathrm{a0}}$	− 19	− 66	− 23

A positive value indicates evidence in favor of the alternative hypothesis.

The results of the hypothesis testing are summarized in Table 4. Following the guidelines of Kass and Raftery (1995) to interpret the results, very strong evidence is found against the three alternative hypotheses. This means that, given the data at hand, it is highly implausible that response times are more alike if they are collected under the same item presentation format. In other words, variation in presentation format does not cause local dependence within the corresponding blocks of items. This result is in line with the very small average effects of the presentation variants on the response times. The effects are extracted from the residuals of the model and indicate that the log-response times are, on average, the lowest in the “Text” variant (“Text” − “Image” $= -$ .025; “Text” − “Text and Image” $=-$ .070) and the highest in the “Text and Image” variant (“Text and Image” − “Image” $=$ .045).

## Discussion

In a novel Bayesian modeling framework, a multivariate generalization of the log-normal response time model has been proposed. The BCSM framework allows the specification of models based on, but not limited to, an integrated likelihood approach. Under the integrated likelihood approach, the random effects are integrated out, and their implied dependencies between observations are directly modeled in a covariance structure in which the random-effect variance parameters serve as covariance parameters. The complexity of the BCSMs is easily controlled, since each random-effect structure is modeled in a separate layer of an additive covariance structure. This is much more difficult in a conditional modeling approach, where each random effect introduces many model parameters and the exact number of parameters depends stronger on the fit of the model.

In the conditional random-effects models, inferences about variance parameters are also problematic. For instance, a random-effect variance of zero can be of specific interest, but the value zero is the lower bound of the corresponding parameter space. The prior specification of a positive variance component can lead to biased parameter estimates and can complicate testing the support for a random effect. In the BCSM, these so-called boundary effects can be avoided, or at least weakened, by extending the parameter space to include negative values. Therefore, shifted inverse-gamma priors are proposed for the variance components, which include a restriction on the parameter space to ensure that the covariance matrix is positive definite but allow negative parameter values, thereby accounting for boundary effects and creating a more exhaustive hypothesis space. Contrary to other priors for variance components such as the half-*t* or half-Cauchy priors, conjugacy is preserved with the proposed truncated shifted inverse-gamma priors. This greatly increases the efficiency of the MCMC sampling algorithm. The proposed priors furthermore lead to less skewness in the posterior distribution if a covariance is close to zero, when compared to priors for variance components. As a result, bias of posterior mean estimates and undercoverage of credible intervals is avoided in a situation where the true value of the (co)variance parameter is located near zero.

The sample size requirements for the BCSM for response times to obtain stable estimates are minimal: for each random-effect structure only two items are needed; this means that the latent speed effect or multidimensional effects (e.g., testlet structure) can be measured with two or more items. In general terms, it is sufficient to have observations from two items to measure an additional dependency, which is modeled as a separate layer in the additive covariance matrix. Furthermore, explicitly modeling each layer of the covariance structure allows testing model assumptions within and across layers. For example, Lee and Neider (2004) point out that in common marginal models it is impossible to test for treatment–random-effect interaction as the marginal models are inferentially identical regardless of whether or not the interaction is present in the corresponding conditional model. In the proposed BCSM framework, these interactions are explicitly modeled and can be tested for, as demonstrated with the testlet structure in the second simulation study and in the context of the empirical example.

The estimates of the BCSM for response times may not be directly comparable to those of a conditional model. This can be caused by different constraints on the parameters space; that is, covariance parameters may take on negative values in the BCSM, while variance parameters in the conditional model have a lower bound at zero. For the above-mentioned reasons, we argue that the (co)variance estimates of the proposed BCSM are more accurate representations of their true values. A related point of caution is the recovery of random effects in the BCSM. As demonstrated in the context of the empirical example, it is possible to recover the random-effect information from the model’s residuals post hoc. Due to the different constraints on the parameter space, the random-effect estimates that are made by the BCSM for response times can be seen as originating from a qualitatively different model, when compared to estimates from a conditional model.

An interesting future prospect of the BCSM for response times is a to combine it with the marginal IRT model by Fox et al. (2017) into a joint-model where the interdependence between response accuracy and speed is explicitly modeled as item-specific cross-covariance parameters. This may lend insight into the effect of, for example different item forms, testlet structures, or time pressure conditions on the speed-accuracy trade-off within a group of persons. Existing approaches to joint-models either assume a constant correlation between response accuracy and speed across persons and items (Glas & van der Linden, 2010; Klein Entink et al., 2008; Loeys, Legrand, Schettino, & Pourtois, 2014; Ranger & Kuhn, 2013; Thissen, 1983; van der Linden & Fox, 2016); do not allow the explicit modeling of the item-specific cross-covariance parameters (Goldhammer & Kroehne, 2014; Goldhammer et al., 2014; Molenaar, Tuerlinckx, & Maas, 2015); or strictly limit the number of states in the speed-accuracy relationship that can be modeled given a reasonable sample size in educational measurement research (Molenaar, Oberski, Vermunt, & Boeck, 2016; Wang & Xu, 2015).

The proposed framework can furthermore be extended with link functions, which translate a latent multivariate normally distributed variable (e.g., response accuracy) into observations that follow a different distribution (e.g., dichotomous item responses). Finally, it is possible to sample directly from the posterior predictive distribution of the data. In the empirical example, this is utilized for proper imputation. However, it also makes the creation of posterior predictive checks (PPC) straightforward. PPCs have been shown to be useful in checking assumptions of IRT models such as multidimensionality or conditional independence (Levy, Mislevy, & Sinharay, 2009) and have been extended to joint-models that incorporate speed and accuracy (Bolsinova & Tijmstra, 2016).

## Appendix A

The sampling steps described below correspond to the sampling scheme as outlined in Algorithm 1. $\varvec{T}^{*} = \{\varvec{T}, \varvec{\omega }\}$ is the imputed dataset.

### Drawing Samples from the Full Joint Posterior

The missing data, item, group, covariance and measurement error variance parameters are iteratively sampled from their respective conditional posterior distribution.

#### Sample Missing Data Parameters

The missing data parameters $\varvec{\omega }$ are sampled from the distribution of the replicated data (i.e., the posterior predictive distribution of the data):

$$\begin{aligned} p(\varvec{\omega }| \varvec{T}, \varvec{R})&= \int p(\varvec{\omega }| \varvec{T}, \varvec{R}, \varvec{\theta })p(\varvec{\theta }|\varvec{T}, \varvec{\omega }, \varvec{R}) \mathrm{d}\varvec{\theta }, \end{aligned}$$

where $\varvec{R}$ are the missing data indicators, $\varvec{\theta }$ is the vector of model parameters, and $p(\varvec{\theta }|\varvec{T}, \varvec{\omega }, \varvec{R})$ is the posterior distribution of $\varvec{\theta }$.

#### Sample Item Parameters

The conditional posterior distribution of the item time intensity parameters is univariate normal with mean $E\left( \lambda _{gk} | \cdot \right)$ and variance $\hbox {Var}\left( \lambda _{gk} | \cdot \right)$:

$$\begin{aligned} E\left( \lambda _{gk} | \varvec{T}^{*}_{gk}, \mu _{\zeta _g}, \sigma _{gk}^2, \delta _g, \mu _{\lambda 0}, \sigma _{\lambda 0}^2\right)&= \hbox {Var}\left( \lambda _{gk} | \cdot \right) \left( \frac{n_g({\bar{T}}^{*}_{.gk} + \mu _{\zeta _g})}{\sigma _{gk}^2 + \delta _g} + \frac{\mu _{\lambda 0}}{\sigma _{\lambda 0}^2}\right) , \\ \hbox {Var}\left( \lambda _{gk} | \sigma _{gk}^2, \delta _g, \sigma _{\lambda 0}^2\right)&= \left( \frac{n_g}{\sigma _{gk}^2 + \delta _g} + \frac{1}{\sigma _{\lambda 0}^2}\right) ^{-1}. \end{aligned}$$

#### Sample Group Parameters

The conditional posterior distribution of each speed group mean parameter is univariate normal with mean $E\left( \mu _{\zeta _{g}} | \cdot \right)$ and variance $\hbox {Var}\left( \mu _{\zeta _{g}} | \cdot \right)$:

$$\begin{aligned} E\left( \mu _{\zeta _{g}} | \varvec{T}^{*}_{g}, \varvec{\lambda }_g, \varvec{\sigma }_{g}^2, \delta _{g}, \mu _{\zeta 0}, \sigma _{\zeta 0}^2 \right)&= \hbox {Var}\left( \mu _{\zeta _{g}} | \cdot \right) \left( \frac{n_{g}({\bar{\lambda }}_g - {\bar{T}}^{*}_{.g.})}{\left( \sum \nolimits _{k=1}^p (\sigma _{gk}^{2}/p + \delta _g)\right) / p} + \frac{\mu _{\zeta 0}}{\sigma _{\zeta 0}^2}\right) , \\ \hbox {Var}\left( \mu _{\zeta _{g}} | \varvec{T}^{*}_{g}, \varvec{\sigma }_{g}^2, \delta _{g}, \sigma _{\zeta 0}^2\right)&= \left( \frac{n_{g}}{\left( \sum \nolimits _{k=1}^p (\sigma _{gk}^{2}/p + \delta _g)\right) /p} + \frac{1}{\sigma _{\zeta 0}^2}\right) ^{-1}. \end{aligned}$$

#### Sample Covariance Parameters

The covariance parameter $\delta _g$ is sampled from its conditional inverse-gamma posterior distribution as defined in Eq. (19). Covariance parameters from additional layers are sampled directly from their conditional posterior that follows from Eq. (22).

#### Sample Measurement Error Variance Parameters

The measurement error variance parameters of the response times $\varvec{\sigma }_{g}^2$ are sampled from their respective conditional inverse-gamma posterior distribution as defined in Eq. (20). The shift parameter and the truncation point are adjusted if layers are added to the covariance matrix.

## Appendix B

Let $\varvec{U}$ have a mean vector equal to $\varvec{0}_p$ (e.g., when $\varvec{U}$ are realizations of the multivariate normally distributed error variances of the model, and thus the residuals). The trace of the diagonalizable covariance matrix, $\hbox {tr}\left( \varvec{\Sigma }_U \right)$, is equal to the sum of its eigenvalues (Axler, 2014, p. 302). In other words, if the basis of the corresponding vector space changes as a result of decorrelating the data (i.e., by multiplying the data matrix with the inverse of the eigenvectors), then the sum of the within sum of squares across all items remains unchanged. To apply this theorem to the problem at hand, first the trace of the CS covariance structure defined in Eq. (16) is derived:

$$\begin{aligned} \hbox {tr}\left( \varvec{\Sigma }_U \right) = (\sigma _{g1}^{2} + \delta _g) + \cdots + (\sigma _{gp}^{2} + \delta _g) = p({\bar{\sigma }}_g^{2} + \delta _g). \end{aligned}$$

Subsequently, let $\varvec{X}$ be the decorrelated data. Furthermore, let the vector $\varvec{Z}_{n_g}$ contain the person means across all items of the transformed data: $\varvec{Z}_{n_g} = \varvec{X}_1 + \cdots + \varvec{X}_p$. From this follows

$$\begin{aligned} Z \sim N(0, p({\bar{\sigma }}_g^{2} + \delta _g)), \end{aligned}$$

whereby the variance thus equals the trace of the covariance matrix as shown in Eq. (5). A truncated inverse-gamma prior that ensures that ${\bar{\sigma }}_g^{2} > 0$ then leads to a posterior truncated shifted inverse-gamma distribution with location parameter $\delta _g$:

$$\begin{aligned} {\bar{\sigma }}_g^{2} \sim IG(\alpha _0 + n_g/2, \beta _0 + \hbox {SSW}/(2p), \delta _g, 0), \end{aligned}$$

where $$\hbox {SSW} = \sum _{i=1}^{n_g} \sum _{k=1}^{p} \left( U_{igk} - {\bar{U}}_{.gk} \right) ^2$$.
